# Human genital antibody-mediated inhibition of *Chlamydia trachomatis* infection and evidence for *ompA* genotype-specific neutralization

**DOI:** 10.1371/journal.pone.0258759

**Published:** 2021-10-18

**Authors:** Caleb M. Ardizzone, Hannah L. Albritton, Rebecca A. Lillis, Caitlyn E. L. Bagnetto, Li Shen, Lisa A. Cavacini, Pamela A. Kozlowski, Alison J. Quayle

**Affiliations:** 1 Department of Microbiology, Immunology, and Parasitology, Louisiana State University Health Sciences Center, New Orleans, Louisiana, United States of America; 2 Division of Infectious Diseases, Department of Medicine, Louisiana State University Health Sciences Center, New Orleans, Louisiana, United States of America; 3 MassBiologics, University of Massachusetts Medical School, Boston, Massachusetts, United States of America; Midwestern University, UNITED STATES

## Abstract

The endocervix, the primary site of *Chlamydia trachomatis* (Ct) infection in women, has a unique repertoire of locally synthesized IgG and secretory IgA (SIgA) with contributions from serum IgG. Here, we assessed the ability of genital and serum-derived IgG and IgA from women with a recent positive Ct test to neutralize Ct elementary bodies (EBs) and inhibit inclusion formation *in vitro* in human endocervical epithelial cells. We also determined if neutralization was influenced by the major outer membrane protein (MOMP) of the infecting strain, as indicated by *ompA* gene sequencing and genotyping. At equivalent low concentrations of Ct EB (D/UW-3/Cx + E/UW-5/Cx)-specific antibody, genital-derived IgG and IgA and serum IgA, but not serum IgG, significantly inhibited inclusion formation, with genital IgA being most effective, followed by genital IgG, then serum IgA. The well-characterized Ct genotype D strain, D/UW-3/Cx, was neutralized by serum-derived IgG from patients infected with genotype D strains, genital IgG from patients infected with genotype D or E strains, and by genital IgA from patients infected with genotype D, E, or F strains. Additionally, inhibition of D/UW-3/Cx infection by whole serum, rather than purified immunoglobulin, was associated with levels of serum EB-specific IgG rather than the genotype of infecting strain. In contrast, a Ct genotype Ia clinical isolate, Ia/LSU-56/Cx, was neutralized by whole serum in a genotype and genogroup-specific manner, and inhibition also correlated with EB-specific IgG concentrations in serum. Taken together, these data suggest that (i) genital IgA most effectively inhibits Ct infection *in vitro*, (ii) human antibody-mediated inhibition of Ct infection is significantly influenced by the *ompA* genotype of the infecting strain, (iii) the genital antibody repertoire develops or matures differently compared to systemic antibody, and (iv) *ompA* genotype-specificity of inhibition of infection by whole serum can be overcome by high concentrations of Ct-specific IgG.

## Introduction

With over 124 million annual global infections, *Chlamydia trachomatis* (Ct) remains the most common sexually transmitted bacterial infection [1]. The greatest burden of disease and pathology is in women of reproductive age, as chronic or repeat infections can lead to pelvic inflammatory disease (PID) and subsequently to infertility [2, 3]. Screening and antibiotic treatment programs can significantly reduce PID rates [4–6], but the lack of access to screening for many women, and a predominantly weeks to months long asymptomatic, or ‘silent’, infection in most, still leaves many women vulnerable. A vaccine is required for mass protection, but advances have been cautioned by the knowledge that pathology is immune-mediated [7]. We therefore believe there is a great need for studies to define what constitutes both a safe and protective immune response to Ct.

Animal models, while limited in recapitulating all elements of Ct infection in the human genital tract, have informed key immune responses likely required for immunity against *Chlamydia* spp. Interferon gamma (IFNγ)-producing CD4+ T cells play a key role in protecting against primary infection with this obligate intracellular pathogen [8–12], while B cells and antibodies are essential for protection against reinfection in murine infection models and provide a degree of protection in guinea pig infection models [12–20]. *In vitro* modeling has also revealed potential mechanisms by which murine antibodies can inhibit *Chlamydia* infection, indicating that antibody isotype, in addition to antigen specificity, dictates functional outcomes. Thus, IgG to the major outer membrane protein (MOMP) of Ct elementary bodies (EB), the infectious extracellular form of Ct, can inhibit or exacerbate infection, but IgA appears to exclusively protect against infection [21, 22].

The primary site of Ct infection in women is the endocervical epithelium. The endocervix is also the major site of antibody production in the female genital tract, and, particularly in chronic infection and inflammation, IgA and IgG plasma cells, and lymphoid follicles and aggregates, can underlie the epithelium [23–27]. In contrast to most mucosal sites, IgG, rather than secretory IgA (SIgA), is the predominant immunoglobulin (Ig) isotype in genital secretions [28]. Studies of genital Ig in hysterectomized women suggest that approximately 50% of the IgG in normal cervicovaginal secretions is derived from passive serum transudate in the vagina [29], where there are few IgG plasma cells [30]. Some of the 50% of IgG derived from the upper tract also likely originates from serum, but a significant proportion appears to be synthesized by local plasma cells in the endocervix [31]. Historic, and our own more recent studies, also suggest that the cervicovaginal antibody repertoire of Ct-infected women is enriched in Ct-specific antibody [32, 33], but the relative neutralizing capacity of serum versus genital anti-Ct antibody, as well as the different isotypes of these antibodies, remains unexplored. This is a critical gap in our knowledge since specific adjuvants, formulations, and delivery strategies for vaccines can influence the type of immunity induced and promote specific antibody isotypes [34]. Human studies are also complicated by the fact that, based on sequence variations in MOMP, there are multiple genotypes of human genital Ct (D-K, with D, E, F, and Ia most prevalent in the US) [35], and both historic human and non-human primate studies indicate protection is serovar-specific [36–39].

Building on our recently described methodology to quantify Ct-specific antibodies [33] and the techniques described here for isolation of genital IgA and IgG, we evaluated the independent ability of genital versus serum IgA and IgG to neutralize EBs *in vitro* and prevent infection of human endocervical epithelial cells. We used samples from a well-characterized cohort of women who recently tested positive for Ct infection, and used additional genital samples to genotype their infecting Ct strain to determine the extent to which the *ompA* (MOMP) genotypes of the infecting strains influence this response.

## Results

### At equivalent concentrations, the Ct neutralizing capacity of genital-derived immunoglobulins is superior to those derived from serum

This study utilized IgG and IgA purified from cervicovaginal lavage (CVL) and serum samples from a cohort of fifty-seven women who were recently diagnosed with genital Ct infection by a standard nucleic acid amplification test (NAAT) (Hologic® APTIMA®) and were returning to the clinic for antibiotic treatment. The cohort was young (median age of 24 years), predominantly African American (77%) and 51% had a history of Ct, as described in previous studies [33, 40].

**Fig 1:**
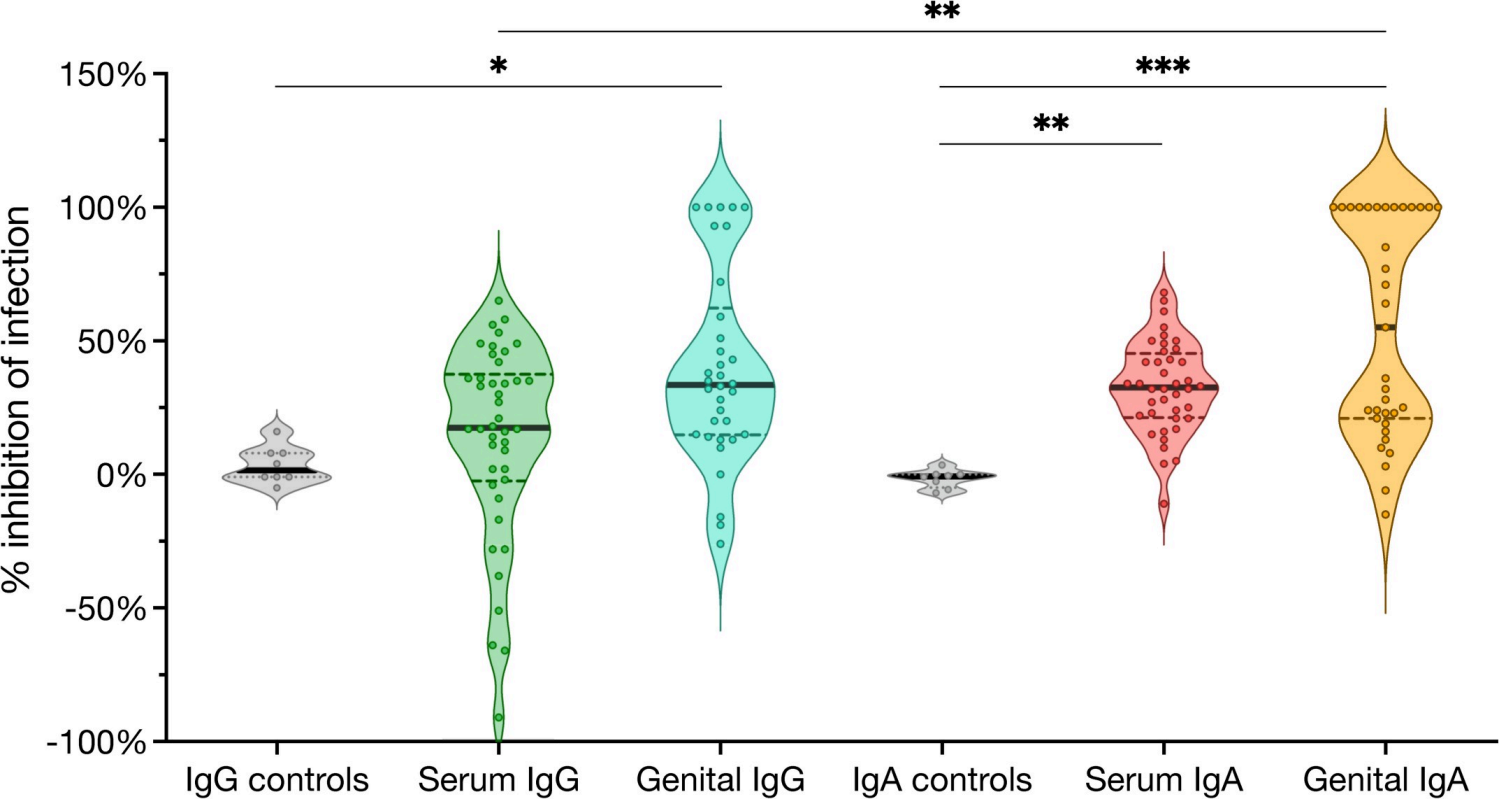
Mucosal antibodies isolated from the genital tract of women diagnosed with Ct infection inhibit *C*. *trachomatis* infection *in vitro*. Percent inhibition of infection of *C*. *trachomatis* D/UW-3/Cx in A2EN cells following incubation with IgG or IgA isolated from serum or CVL of patients diagnosed with *C*. *trachomatis* infection. IgG and IgA were tested at 5 ng/ml concentrations of anti-Ct antibody. Horizontal bars represent mean percent inhibition of infection. Kruskal-Wallis test followed by Dunn’s post-test, *p<0.05; **p<0.01; ***p<0.001.

We first evaluated the capacity of CVL- and serum-derived antibodies from each woman to inhibit *in vitro* Ct infection of human endocervical epithelial cells. We used whole-EB and total immunoglobulin (Ig) ELISAs, previously used to characterize the first twelve patients of this cohort [33], to determine (i) anti-Ct IgG and IgA and (ii) total IgG and IgA concentrations in serum, vaginal secretions, and endocervical secretions for the remainder of the cohort (n = 45) to calculate EB specific activity (ng anti-EB IgG or IgA antibody per μg total IgG or IgA, respectively) (S1 Fig). IgG and IgA were then isolated from matched serum and concentrated CVL from each patient, and total IgG and IgA concentrations were again measured. EB-specific antibodies in the IgG and IgA preparations purified from CVL were not measured due to their limited amount. Instead, levels of EB-specific antibodies in the CVL-derived IgG or IgA were approximated by averaging the EB specific activity of IgG or IgA in the subject’s vaginal and cervical secretions, and estimating based on the total IgG or IgA concentrations measured in the CVL-derived IgG and IgA. Based on the EB specific activity, purified IgG and IgA were diluted to contain an equivalent concentration of EB-specific antibody. Neutralizing activity was then assessed against the well-characterized genital *C*. *trachomatis* strain D/UW-3/Cx by co-incubation of Ig with purified EBs and enumeration of inclusion forming units (IFU) after inoculation of A2EN endocervical epithelial cell monolayers. At equivalent concentrations of anti-Ct antibodies, serum IgA, genital IgG, and genital IgA significantly inhibited Ct inclusion formation *in vitro* compared to isotype controls, but serum IgG did not. Genital IgA most strongly inhibited Ct infection, and only these samples achieved a mean percent inhibition of greater than 50% (Fig 1). Taken together, these data suggest that IgG and IgA isolated from genital secretions are more inhibitory against *C*. *trachomatis* infection than IgG and IgA isolated from serum, and suggest that there are functional differences in antibody repertoires by both isotype and site.

### Genital IgG and IgA inhibit Ct infection in a genotype and genogroup-specific manner

The kernel distribution of mucosal-derived IgG and IgA data (Fig 1) visualized two distinct patient populations: those that significantly neutralized infection, and those that did not. Given that historic human and non-human primate trachoma studies suggest that immunity to Ct is serovar specific [37, 38], we next determined if this differential inhibition could be due to differences in the antigen specificity of patient antibodies. Since MOMP, encoded by *ompA*, comprises the majority of the protein mass of the outer membrane of EBs and is established an immunodominant antigen, we hypothesized that patient antibodies would most efficiently inhibit infection by Ct strains matching that of the current infecting *ompA* genotype or genogroup.

**Fig 2:**
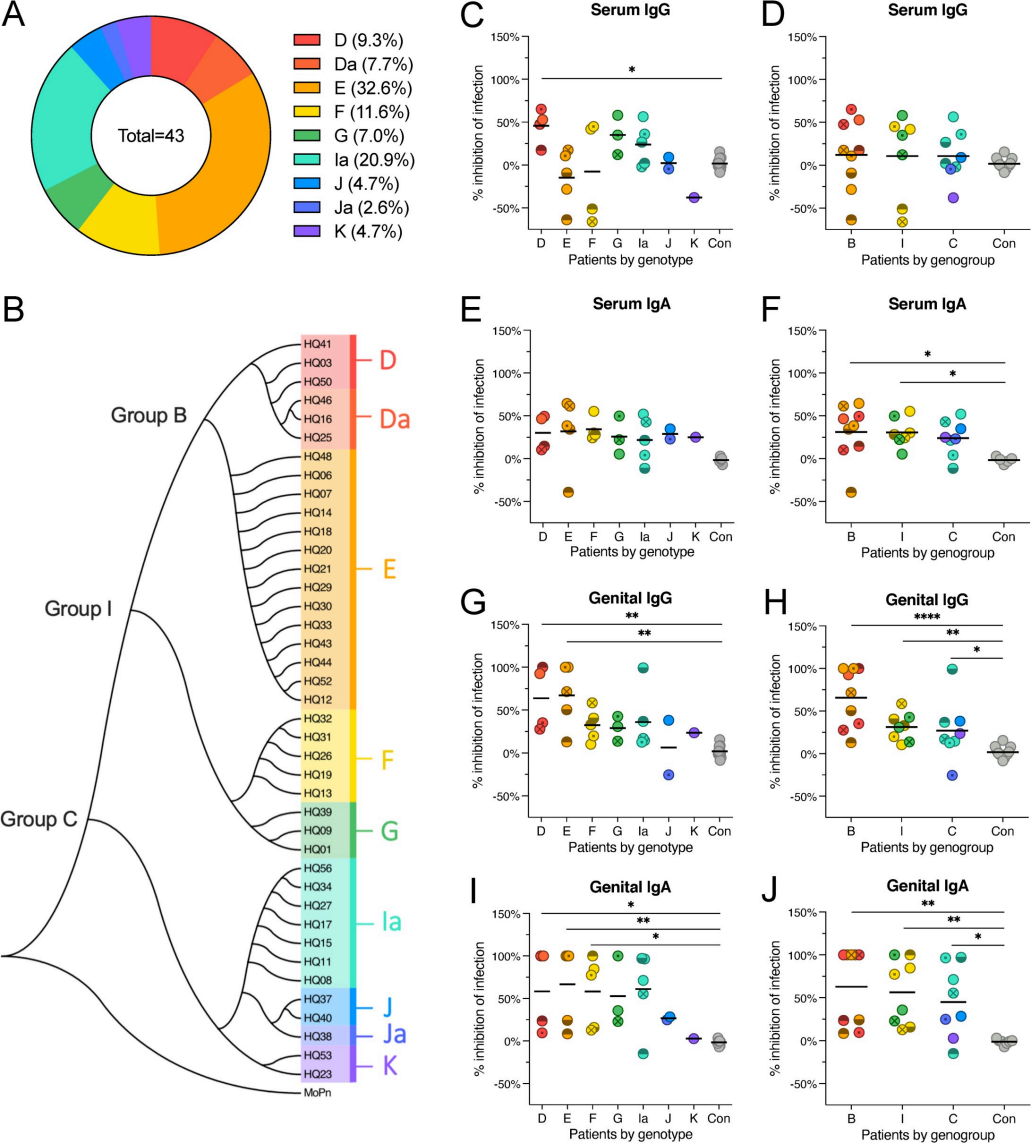
Genital antibody-dependent inhibition of *C*. *trachomatis* infection is influenced by the genotype and serogroup of the infecting strain. (A) Relative abundance of *ompA* genotypes. (B) Consensus phylogenetic tree of aligned *ompA* gene sequences amplified from *C*. *trachomatis* clinical isolates. Numbers on tree represent individual patients. Letters on tree (Sv D-K, Da, Ia, Ja) indicate the genotype of each cluster of clinical isolates. (C-J) Percent inhibition of infection of *C*. *trachomatis* D/UW-3/Cx in A2EN cells following incubation with IgG or IgA purified from serum or CVL of patients diagnosed with *C*. *trachomatis* infection. (C, E, G, I) Patients grouped by genotype of infecting strain. (D, F, H, J) Patients grouped by serogroup of infecting strain. Points represent individual patients. Grey dots indicate isotype controls. Bars represent mean percent inhibition of infection. (C-J) Kruskal-Wallis test followed by Dunn’s post-test, *p<0.05; **p<0.01; ***p<0.001, ****p<0.0001.

First, to identify infecting genotypes, we amplified the *ompA* gene by PCR using DNA isolated from genital swabs (S1 Table) [41, 42]. All Ct samples from NAAT-positive, culture-positive patients were successfully genotyped. Nine unique *ompA* sequence types were identified, including three *ompA* genotype variants (Da, Ja, and Ia). The distribution of *ompA* genotypes was E (33%), I/Ia (21%), D/Da (17%), F (12%), G (7%), J/Ja (8%), and K (5%) (Fig 2A). These *ompA* genotypes clustered into three distinct genogroups, corresponding to previously described serogroups B (serovars D/Da, E), intermediate (I) (serovars F, G), and C (serovars I/Ia, J/Ja, K) (Fig 2B) [43–47].

To determine if antibodies were directed primarily against the infecting strain, IgG and IgA data from paired serum and CVL were then grouped by genotype and genogroup and neutralizing activity against D/UW-3/Cx was evaluated. Serum-derived IgG from patients infected with genotype D strains significantly inhibited inclusion formation of D/UW-3/Cx, but no genogroup-specific neutralization was observed (Fig 2C and 2D). Serum IgA failed to exhibit genotype-specific neutralization, and although IgA isolated from serum of patients infected with genogroup B strains significantly neutralized D/UW-3/Cx compared to isotype controls, the mean inhibition did not meet the threshold of 50% reduction in infection (Fig 2E and 2F). CVL-derived IgG from patients infected with *C*. *trachomatis* strains D/Da or E significantly inhibited infection by D/UW-3/Cx compared to isotype controls, and while significant genogroup-specific inhibition was observed for all genogroups, only genogroup B surpassed the 50% threshold for inhibition (Fig 2G and 2H). Genital IgA from patients infected with genotypes D/Da, E, and F inhibited D/UW-3/Cx infection, and while significant genogroup-specific inhibition was observed for all genogroups, only genogroups B and I met the 50% threshold (Fig 2I and 2J). Taken together, these data indicate that patient-derived antibodies significantly inhibit inclusion formation by Ct *ompA* genotypes similar to the current infecting strain and that genital IgA is more broadly reactive against a wide range of Ct *ompA* genotypes. These results suggest that patient-derived antibodies are primarily, but not necessarily, directed against the infecting Ct strain.

### Inhibition of Ct infection by serum is dependent on antibody concentration rather than genotype- or genogroup-specific activity

**Fig 3:**
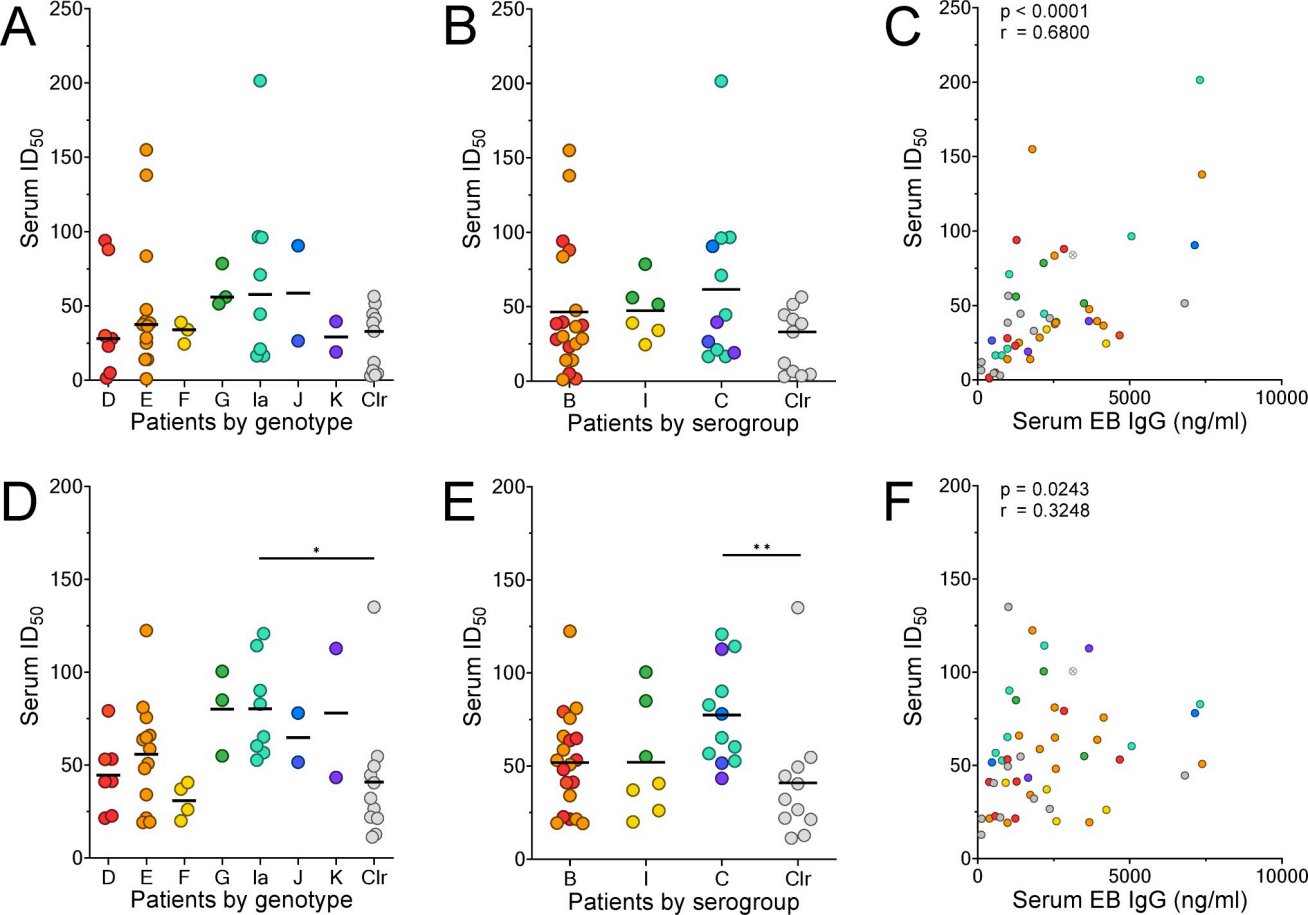
Serum antibody-dependent inhibition of *C*. *trachomatis* infection is influenced by both EB-specific IgG concentration and *ompA* genotype of the infecting strain. (A) Serum neutralizing antibody titers (ID_50_) to D/UW-3/Cx with patients grouped by infecting serovar. (B) Serum neutralizing antibody titers to D/UW-3/Cx with patients grouped by serogroup of infecting strain. (C) Scatter plot of serum ID_50_ values against D/UW-3/Cx plotted against concentrations of EB-specific IgG in patient serum. (D) Serum ID_50_ values against Ia/LSU-56/Cx with patients grouped by serovar of infecting strain. (E) Serum ID_50_ values against Ia/LSU-56/Cx with patients grouped by serogroup of infecting strain. (F) Scatter plot of serum ID_50_ values against Ia/LSU-56/Cx plotted against concentrations of EB-specific IgG in patient sera. Points represent individual patients. Grey dots indicate patients that spontaneously cleared *C*. *trachomatis* infection. The point with an "X" denotes a patient for which the *ompA* genotype could not be determined. Dotted lines denote starting serum dilution. (A,B,D,E) Kruskal-Wallis test followed by Dunn’s post-test, *p<0.05; **p<0.01; ***p<0.001. (C,F) Spearman’s rank-order correlation.

While the previous experiment was useful in identifying potential differences in the neutralizing activity of patient-derived antibodies by isotype, site derivation, and *ompA* genotype of the infecting strain, the assay did not evaluate dose-dependent inhibition or Ct strains other than D/UW-3/Cx belonging to genogroup B. To address this limitation, we isolated and expanded a genotype Ia and genogroup C Ct sample, Ia/LSU-56/Cx, from a patient swab and developed a serum neutralization assay using *C*. *trachomatis* D/UW-3/Cx or Ia/LSU-56/Cx infection of A2EN cells. We hypothesized that serum from patients infected with genotype D or genogroup B strains would significantly inhibit D/UW-3/Cx infection, while serum from patients infected with genotype Ia or genogroup C strains would significantly inhibit Ia/LSU-56/Cx. Differences in serum ID_50_ against D/UW-3/Cx were not due to the genotype or genogroup of the infecting strain (Fig 3A and 3B); however, a strong correlation was observed between serum ID_50_ and the concentration of EB-specific IgG in serum measured in the whole-EB ELISA (Fig 3C). Conversely, serum from patients infected with Ct strains belonging to genotype Ia or genogroup C significantly inhibited infection by Ia/LSU-56/Cx compared to serum from patients who had cleared Ct infection (Fig 3D and 3E), and a significant correlation was found between serum neutralization titers (ID_50_) and the concentration of EB-specific IgG in serum, although this correlation was weaker than that observed against D/UW-3/Cx (Fig 3F). Taken together, these data suggest that inhibition of Ia/LSU-56/Cx by sera is determined by both the *ompA* genotype of the infecting strain and the amount of EB-specific IgG, while inhibition of D/UW-3/Cx is influenced primarily by the amount of EB-specific IgG rather than the *ompA* genotype of the infecting strain.

## Discussion

With current Ct vaccine initiatives, but minimal functional information on the human genital anti-Ct antibody repertoire, we developed methodology to evaluate the neutralizing activity of antibodies harvested from the female genital tract. Using samples from women recently diagnosed with a Ct infection we compared the relative effectiveness of IgG and IgA, potential differences in serum and genital secretion-derived antibody, and the ability of antibody to neutralize common serovars of infecting Ct strains. By investigating these parameters, we were able to reveal the individual ability of IgG and IgA to neutralize Ct, how site of origin influences function, and recreate classic studies that suggest immunity to Ct infection is strain-specific.

Clear differences were observed in the ability of antibody of specific isotypes and site derivations to neutralize Ct *in vitro*. At equal low concentrations, genital-derived EB-specific antibody more efficiently neutralized Ct compared to antibody from serum, and only antibodies from genital secretions completely inhibited Ct infection. This suggests that the genital anti-Ct antibody repertoire may have higher affinity and/or be more broadly reactive, and it is reasonable to presume that these antibodies are derived from local plasma cells in the cervix [27, 48].

Genital IgA was superior for neutralizing *in vitro* Ct infection and was the only antibody isotype for which the mean inhibition of infection surpassed 50%. This might be due to higher avidity of the polymeric IgA molecule compared to monomeric IgG [49]. This may also be of significance as IgG and IgA could compete *in vivo* and greatly differ in proportion to each other, with IgG at roughly 2–4 fold higher concentrations than IgA in female genital secretions [50–53]. Immunoglobulin isotypes significantly differ in function, and SIgA is a first line of adaptive immunity against many mucosal pathogens [30, 54, 55]. Anti-MOMP SIgA inhibits Ct infection *in vivo* and *in vitro* in murine models, and anti-Ct SIgA inversely correlates with cervical infectious Ct burden in humans [22, 32]. In contrast, while *in vivo* murine models indicate that anti-MOMP and anti-LPS IgG can inhibit Ct infection and reduce shedding in mice [19], *in vitro* opsonization of EBs by some monoclonal IgG antibodies can enhance EB uptake *in vitro* by epithelial cells expressing FcγRII, FcγRIII, or FcRn [22, 56, 57], illustrating potentially more dichotomous roles for IgG. While it is currently unknown whether A2EN express these surface FcγRs, we observed that purified serum IgG from 10% of our cohort significantly enhanced Ct infection in our neutralization assays (>150% infection compared to untreated controls), and these patients were infected with Ct genotypes other than genotype D. Future studies will be required to determine if the A2EN cell line express surface FcγRs capable of mediating the uptake of IgG immune complexes. Although serum IgG from only a small number of patients exacerbated Ct infection *in vitro*, these results, taken together, suggest that IgA may be the safest and most effective neutralizing isotype to be induced by a Ct vaccine.

Genital IgG and IgA samples from a subset of patients completely protected against *in vitro* Ct infection, while the remainder exhibited minimal inhibition, suggesting differences in the antigen specificity of their respective antibody repertoires. Since the majority, over 60%, of the protein mass of the outer membrane of EBs is comprised of MOMP [58], an immunodominant and antigenically diverse surface protein encoded by the *ompA* gene, we chose to focus on this surface antigen. Variation in MOMP between *Chlamydia* strains was the basis for the identification of 19 distinct serovars by monoclonal antibodies targeting various strain-specific epitopes within the protein [44, 59], and these serovars are now classified as *ompA* genotypes through the widespread use of PCR and DNA sequencing [60–65]. Since a Ct serovar D strain, D/UW-3/Cx, was utilized in the early neutralization assays, we hypothesized that D/UW-3/Cx infection would be more efficiently inhibited by genital antibodies from patients infected with identical or similar Ct genotypes. Genital IgG and IgA isolated from patients infected with genotype D and E (genogroup B) strains most efficiently inhibited D/UW-3/Cx infection in comparison to antibodies from patients infected with other genotypes. Genital IgA was more broadly protective than genital IgG, greatly inhibiting Ct infection over a larger range of genotypes and genogroups, similar to observations made in neutralization studies of heterologous influenza viruses by vaccine-induced respiratory IgA [66–69]. While strain-specific humoral responses have been documented in human and non-human primate ocular *Chlamydia* infection, we are unaware of any studies investigating these strain-specific responses in the human genital tract. By genotyping the genital Ct strains infecting patients in our cohort, we were able to determine whether antibodies from those patients more effectively inhibited identical or similar Ct genotypes. Re-infection experiments by others showed that antibody responses are narrowly directed against the MOMP of the infecting serovar, which broadens over time to include cross-reactive antibodies until the infection is cleared [39]. Interestingly, antibodies against the serovar of the primary infection were recalled to a higher level during secondary infection with a different serovar, and antibody responses were directed mainly against the original infecting serovar, even after re-infection with an identical or different serovar [37, 38]. Taken together, the results of historic and our own studies suggest that the serovar of the infecting strain can shape the humoral immune response.

For a vaccine to generate genital Ct-specific IgA antibodies in genital secretions, nasal or vaginal immunization would likely be optimal, and the former would be particularly advantageous because, in contrast to vaginal immunization, nasal immunization generates a robust genital immune response and serum IgG antibodies [70]. This could be important as at least half of the IgG present in the female genital tract is transudate from serum. The information gained from this study could inform STI vaccine designs since route of delivery, formulation, and choice of adjuvant can modulate isotype bias and the induction of mucosal versus systemic immunity [34]. Since vaccine-induced circulating IgG contributes to the genital antibody repertoire, it is important to determine whether serum antibody in Ct-infected patients is also capable of neutralizing Ct and whether this protection is genotype specific. Through evaluation of serum neutralization titers against multiple Ct genotypes, including a Ct serovar Ia strain, Ia/LSU-56/Cx, we determined that neutralization of D/UW-3/Cx and Ia/LSU-56/Cx by whole serum was determined primarily by the concentration of EB-specific IgG, and that Ia/LSU-56/Cx, but not D/UW-3/Cx, was neutralized most by patients infected with genotype Ia strains. Taken together, these results suggest that Ct-specific antibodies in serum are less effective and less broad in reactivity than those in genital secretions. However, serum antibodies are still able to inhibit Ct infection, albeit at much higher concentrations than those required for neutralization by genital IgG and IgA.

One limitation of this study is the focus on neutralization, since previous murine studies indicate the role of B cells in anti-Ct immunity is likely much broader (reviewed in [48, 71]). Classic murine studies by Morrison et al. demonstrated that B cells shape cellular immunity through Ct antigen presentation [72–74], and that antibody cooperates with IFNγ-activated cell populations to enhance killing of Ct [20]. We should also note that although studies in mice have shown that Ct infection-induced antibodies can prevent dissemination of Ct, a recent human study reported that, while serum and cervical anti-Ct antibody inversely correlated with Ct genome copy number, Ct genomes could still be detected in the endometrium of women with these antibodies [75]. While complex to undertake, additional studies of B cells and antibodies in Ct-infected women should be performed, especially in the context of restricting Ct infection in the complex human genital milieu, and distinguishing infectious from noninfectious Ct.

If the outcome of an immune response to Ct is dependent on the *ompA* genotype, this would also have implications for interpretation of both natural history studies and Ct vaccination strategies. A Ct vaccine currently in clinical trials is an intramuscular prime/nasal boost multivalent MOMP vaccine based on genogroups B and I, including genotypes D-F, and excludes genogroup C (genotypes I-K) [76]. Our data indicate that antibodies most effectively neutralize Ct genotypes that are identical or similar to the Ct strain infecting the patient, and cross-neutralization of Ct strains from multiple genogroups by genital IgA shows that induction of IgA responses in the female genital tract may be ideal for protection against a broad range of Ct strains. Taken together, our data suggest that vaccine-induced humoral responses to MOMP may not elicit mucosal antibodies that effectively neutralize serogroup C strains. While the vaccine may protect most women against Ct infection, a significant proportion of our study cohort (~20%) were infected with genotype Ia strains (Fig 2A), and it is currently unknown if the vaccine will be effective against Ct strains belonging to genogroup C. However, it is important to note that our study only evaluated humoral immune responses following natural infection with different Ct genotypes, not vaccine-induced immune responses, which may differ.

In summary, this study indicates that IgA in genital secretions broadly neutralizes Ct genotypes across multiple genogroups, and that antibodies derived from genital secretions are functionally different from those in serum. Future studies and vaccine trials should investigate genital antibodies in addition to those in serum, and it is important that Ct vaccine designs consider the impact of MOMP specificity on the neutralizing capacity of vaccine-induced antibodies against multiple Ct genotypes, particularly genogroup C strains. The methodology for culturing Ct isolates from patients in our cohort can be expanded for use in future studies and clinical trials, offering the unique opportunity to test patient antibodies against their own Ct isolate or identical/similar genotypes circulating in the same population.

## Methods

### Participants, specimen collection, and STI testing

Fifty-seven women aged 18–35 years were recruited from the Louisiana State University (LSU) CrescentCare Sexual Health Center in New Orleans, LA. Women were eligible for the study if they were returning to the clinic for azithromycin treatment and counseling after a recent (<1 month) positive CT GENPROBE® APTIMA® nucleic acid amplification test (NAAT). The study was approved by the Louisiana State University Health Sciences Center—New Orleans Institutional Review Board and written consent was obtained from each patient. Detailed demographics of this cohort have been reported previously, and concentrations of total and EB-specific IgG and IgA in serum and genital secretions for the first twelve patients of this cohort have been published previously [33, 40]. Informed consent was obtained from each patient. Serum, vaginal and endocervical secretions, and 5 ml of cervicovaginal lavage (CVL) were collected as previously described [33]. *Chlamydia trachomatis*, *Neisseria gonorrhoeae*, and *Trichomonas vaginalis* were detected by the APTIMA® Combo 2 test, bacterial vaginosis by Amsel and Nugent scoring, and viable Ct cervical burden was determined by semi-quantitative culture for inclusion forming units (IFU; median of 878,596 IFU per swab) [40, 42].

### Isolation of *C*. *trachomatis* Ia/LSU-56/Cx

To promote efficiency of Ct isolation and expansion from an endocervical swab, cell cycle arrest of McCoy B fibroblast cells (CRL-1696, ATCC) was performed as previously described with several modifications [77]. Briefly, McCoy cells were grown in Eagle’s Minimum Essential Medium (EMEM) supplemented with 10% serum and 1X GlutaMAX until 90% confluent. Monolayers were treated with 1 mM thymidine for 24 hours, washed with DPBS, then treated with 1 μg/ml mitomycin-C for 16 hours. Cell monolayers were then washed and allowed to rest overnight. Cells were then split 1:2 and incubated overnight. G2/M-arrested McCoy cells were seeded onto 24-well plates (2x10^4^ cells per well). To process the Ct containing transport medium, sterile 3 mm glass beads were added to each tube, which was then vortexed for one minute and rested on ice for one minute, three times. Monolayers were inoculated with 150 μl processed Ct transport medium and centrifuged at 1,800 RCF for 40 minutes at 25°C. Inoculum was removed and replaced with Ct infection medium (McCoy growth medium supplemented with NaHCO_3_, HEPES, glucose, and an antibiotic cocktail composed of gentamycin, vancomycin, nystatin, and amphotericin B), then monolayers were incubated at 37°C in 5% CO_2_. Cells were examined every 8–10 hours, and cells and media were collected together when large, mature inclusions were visible then passaged onto fresh monolayers of G2/M-arrested McCoy cells for further expansion.

### Purification of *C*. *trachomatis* elementary bodies

Ct D/UW-3/Cx and E/UW-5/Cx EBs for whole-EB ELISAs were purified as previously described [33, 78]. Ct D/UW-3/Cx and Ia/LSU-56/Cx EBs for neutralization assays were purified using a modified protocol optimized for a high yield of infectious EBs [78]. Briefly, confluent monolayers of L929 fibroblast cells (CCL-1, ATCC) were inoculated with infected cell lysate (MOI 2), incubated for three days, then harvested. EBs were purified from cell lysate using ultracentrifugation with an OptiPrep cushion gradient (34%) in sucrose phosphate glutamate buffer (SPG) (10 mM sodium phosphate [8 mM Na_2_HPO_4_−2 mM NaH_2_PO_4_], 220 mM sucrose, 0.50 mM L-glutamic acid), followed by two washes with SPG.

### Total immunoglobulin ELISA

Concentrations of total IgG and IgA in serum and genital secretions were measured using a previously described ELISA [79]. Briefly, plates were coated with affinity-purified goat anti-human IgG γ chain (ICN reagents) or IgA α chain-specific antibodies (MP Biomedicals) in PBS. Plates were washed with PBS containing 0.05% Tween in PBS (PBST) and blocked with 2% goat serum (GS) (Equitech-Bio Inc) in PBST. Plates were loaded with serial dilutions of test samples and calibrated IgG or serum IgA reference standards [79]. Then, plates were treated with biotinylated affinity-purified goat anti-human IgG γ chain or IgA α chain-specific antibodies (Southern Biotech), reacted with neutralite avidin-labeled horseradish peroxidase (Southern Biotech), and developed with tetramethylbenzidine (Southern Biotech). Absorbance was recorded at 370 nm on a SpectraMax plate reader (Molecular Devices), and immunoglobulin concentrations were interpolated from 4-parameter standard curves calculated in SoftMaxPro (Molecular Devices).

### Whole-EB ELISA

Concentrations of Ct EB-specific IgG and IgA in serum and secretions were measured using a previously described whole-EB ELISA [33]. Briefly, plates were coated overnight with poly-L-lysine. An equal number of purified D/UW-3/Cx and E/UW-5/Cx EBs were then added and the plate was centrifuged to facilitate contact of the EBs with the poly-L-lysine. After brief fixation with glutaraldehyde, the plate was blocked and serially dilutions of standard and samples were added. After overnight reaction at 4˚C, plates were washed and developed as described above. Concentrations of Ct EB-specific IgG or IgA in each sample were interpolated from four-parameter standard curves constructed using serum IgG and IgA standards [33] obtained from pooled serum of women diagnosed with Ct infection as IgA or IgG standards.

### Isolation of IgG and IgA from clinical specimens

Total IgA and IgG was purified from CVL that had been concentrated using Amicon Ultra 15 centrifugal filters and from serum using Peptide M agarose (InvivoGen) and recombinant Protein G (PG) sepharose (GE Healthcare), respectively. Isolation was done by placing 500 μl of a 10% Protein G slurry in PBS in the upper chamber of a 0.45 μm SpinX cellulose acetate microfuge tube (Costar), then centrifuging the tube at 15,000 RCF for 1 minute to remove the PBS. The dry PG in the upper chamber was immediately reconstituted with serum or CVL and placed in a new lower chamber. After 1 hour of mixing, the tube was centrifuged and the IgG-depleted fluid in the lower chamber was placed on ice for later isolation of IgA. The PG-bound IgG in the upper chamber was washed 2 times with PBS. The IgG was then eluted into the lower chamber by adding 100 μl of 0.1 M glycine, pH 2.5, and centrifuging after 1 minute, then repeating this procedure. The 200 μl of IgG in the lower chamber was immediately neutralized using saturated Tris base. The IgA in the initial flow-through was then similarly purified using a 20% slurry of Peptide M. The purified IgG and IgA preparations were dialyzed overnight in 500 ml of PBS at 4°C, then sterile-filtered using sterile 0.22 μm SpinX cellulose acetate microfuge tubes (Costar) and stored at 4°C.

### *C*. *trachomatis* neutralization assay using purified IgG or IgA

The immortalized human endocervical epithelial cell line, A2EN, was used for all neutralization studies [80, 81]. A2EN cells were seeded in 96-well flat, transparent bottom black plates at 2 x 10^4^ cells/well and maintained in keratinocyte serum-free medium (KSFM; supplemented with human recombinant epidermal growth factor (rEGF), bovine pituitary extract (BPE), 40 ng Ca^2+^, and 1X GlutaMAX; Thermo-Fisher Scientific) until 90% confluent. Purified IgG and IgA from serum and CVL were utilized in this series of experiments. Genital IgG and IgA were diluted to an equivalent concentration of 5 ng/ml of EB-specific antibody per sample and tested in triplicate; serum IgG and IgA were used at 5 ng/ml and tested in duplicate. Thirty-five μl of sample was added to D/UW-3/Cx EBs and incubated at 37°C on a shaker incubator at 110 RPM for 1 hour. A2EN cell media was aspirated and replaced with 30 μl of Ig-EB mixture. EBs for Ct infected only controls were incubated in SPG. Plates were centrifuged for 40 minutes at 1800 RCF at 25°C. After centrifugation, Ig-EB mixture was aspirated and replaced with KSFM. Cells were incubated at 37°C for 48 hours after which medium was aspirated and cells were fixed with 4% formaldehyde for 15 minutes, washed with PBS, and permeabilized with equal parts methanol and acetone for 20 minutes at -20°C. Cells were blocked with a Background Sniper (Biocare Medical) at room temperature for one hour. Chlamydial inclusions were visualized with Merifluor anti-chlamydial lipopolysaccharide (LPS) antibody conjugated to fluorescein isothiocyanate (FITC), which contains Evans Blue as a counterstain, for 30 minutes (Meridian Bioscience), and 4,6-diamidino-2-phenylindole (DAPI; Molecular Probes) was used to visualize nuclei. PBS was added to wells and plates were kept at 4°C. Uninfected A2EN cells and Ct infected cells without antibodies were performed in triplicate for each plate. Inclusions and nuclei were visualized at 20X using a Zeiss Observer.Z1 microscope, and triplicate pictures of each sample were captured using ZEN 2.3 Pro software. Nuclei were enumerated using CellProfiler 3.1.5, and inclusions were enumerated using ImageJ 2.0.

The following human IgG monoclonal antibodies and myeloma proteins were used as negative controls at 5–50 ng/ml concentrations in triplicate in three separate experiments: clone 4006 anti-*Escherichia coli* heat-labile toxin (LT) [82] and F240 anti-HIV-1 gp41 [83] (from Dr. Lisa Cavacini), 23.1D anti-Lassa virus IgG [84], 2.2B anti-HIV-2 gp41 and 7B2 anti-HIV-1 gp41 [85] (kindly provided by Dr. James E. Robinson, Tulane), F105 anti-HIV-1 gp120 (from the NIH AIDS Reagent Program) [86, 87], and two IgG myeloma proteins (from Dr. Jiri Mestecky, University of Alabama at Birmingham). The following were used as IgA negative controls: monomeric IgA1 myeloma protein, colostral IgA (Sigma) and clone 4006 isotype-switched recombinant anti-LT monomeric IgA1 and IgA2, dimeric IgA1 and IgA2, and SIgA1 and SIgA2 antibodies, produced as described [82].

### *ompA* genotyping

DNA extraction from endocervical and vaginal swab specimens was performed using the DNeasy Blood & Tissue Kit (Qiagen) following the manufacturer’s instructions. DNA samples were initially amplified for the Ct *ompA* gene by PCR using the conditions described by Ficarra et al. [42]. Samples that failed to amplify *ompA* under these conditions were amplified using the conditions described by Lysen et al. [41]. Samples that failed to amplify under either of these conditions were amplified under new conditions using primers OmpSeqF and OMP-N2R (5’-CTGCGTATTTGTCTGCATCRA-3’) followed by OMP-N2F (5’-CAGCATGCGTGTTGGTTACT-3’) and OmpSeqR [88]. Each PCR reaction comprised of 500 nM forward and reverse primers, 1X Q5 High- Fidelity DNA Polymerase Master Mix (New England Biolabs), and 1 ng-1 μg DNA extract. Amplification was carried out on a MJ Mini Thermal Cycler (Bio-Rad), with the following conditions: initial denaturation at 98°C for 30 seconds, then 5 cycles of 98°C for 10 seconds, 30 seconds at 56°C, and 30 seconds at 72°C, followed by a final extension at 72°C for 2 minutes. If no product was obtained after attempted amplification with other primers and the sample was culture positive, then DNA was extracted from *in vitro* culture and amplified. Amplicon size was confirmed by agarose gel electrophoresis, then purified using QIAquick PCR Purification Kit (Qiagen) according to manufacturer’s instructions. Purified PCR products were sequenced by Eurofins Genomics (Louisville, KY). Sequences were trimmed to exclude segments with quality scores below 25 using SnapGene (GSL Biotech), and each sequence was then aligned with published *ompA* sequences by BLASTn to determine the *ompA* genotype.

### Phylogenetic analysis

Trimmed Ct *ompA* sequences were aligned by Clustal Omega. The phylogenetic tree was constructed from a distance matrix of taxa using the neighbor-joining method in MEGA7 [89]. The consensus tree was generated following 1,000 iterations of the analysis. The murine *Chlamydia* strain, *C*. *muridarum* (MoPn), was used as an outgroup in all analyses.

### *C*. *trachomatis* neutralizing assay with serum

A2EN human endocervical epithelial cells [80] were seeded and maintained in 96 well plates until 90% confluent, as described above. Serum was heat-inactivated by a 30 minute incubation at 56°C, diluted 1:10 and filter sterilized using Spin-X 0.22 μm cellulose acetate microfuge tubes (Costar). In a 96-well V-bottom plate, serial two-fold dilutions of serum were made in sucrose phosphate glutamate buffer (SPG), mixed with D/UW-3/Cx or Ia/LSU-56/Cx Ct EBs, then incubated for 1 hour at 37°C at 110 RPM on an Excella E25 shaker incubator. Culture medium was aspirated from cell monolayers, replaced with 50 μl of serum-EB mixture, then plates were centrifuged at 1800 RCF for 40 minutes at 25°C. After centrifugation, serum-EB mixtures were aspirated and replaced with fresh KSFM, then plates were incubated for 60 hours at 37°C. Cell monolayers were fixed and permeabilized as described above. Inclusions were visualized using mouse monoclonal antibody to *Chlamydia* genus-specific LPS (clone FIA6) [90] and an anti-mouse IgG Alexa Fluor 488 secondary antibody (LifeTech). Nuclei were stained as described above, and monolayers were covered in 50 μl Tris-buffered glycerol containing p-phenylenediamine. Inclusions and nuclei were visualized using a BioTek Cytation™1. Nine images per well were captured at 20X, and nuclei and inclusions were enumerated using BioTek Gen5 software. ID_50_ values were calculated using GraphPad Prism software and nonlinear regression analysis.

### Statistical analyses

Statistical analyses were performed using Prism (v9.1.1; GraphPad, San Diego, CA). Kruskal-Wallis test followed by Dunn’s test was used to compare results in most experiments. Spearman’s rank-order correlation was used to measure the strength and direction of association between EB-IgG concentrations and serum ID_50_ values against D/UW-3/Cx and Ia/LSU-56/Cx. P-values greater than 0.05 were considered significant.

## Supporting information

S1 Table Primers used for the amplification and sequencing of the *ompA* gene. (XLSX)

S1 Fig IgG and IgA in vaginal and cervical secretions of Ct-infected women as measured by total and whole-EB ELISAs. EB specific activity was calculated by dividing Ct-specific antibody by total immunoglobulin for each isotype and sampling site (ng anti-EB IgG or IgA antibody per μg total IgG or IgA, respectively).
(TIF)
