# Supplementary figures and images for: Human genital antibody-mediated inhibition of Chlamydia trachomatis infection and evidence for ompA genotype-specific neutralization
Source: PLoS One. 2021 Oct 18;16(10):e0258759. doi: 10.1371/journal.pone.0258759 (PMC8523062; doi:10.1371/journal.pone.0258759)

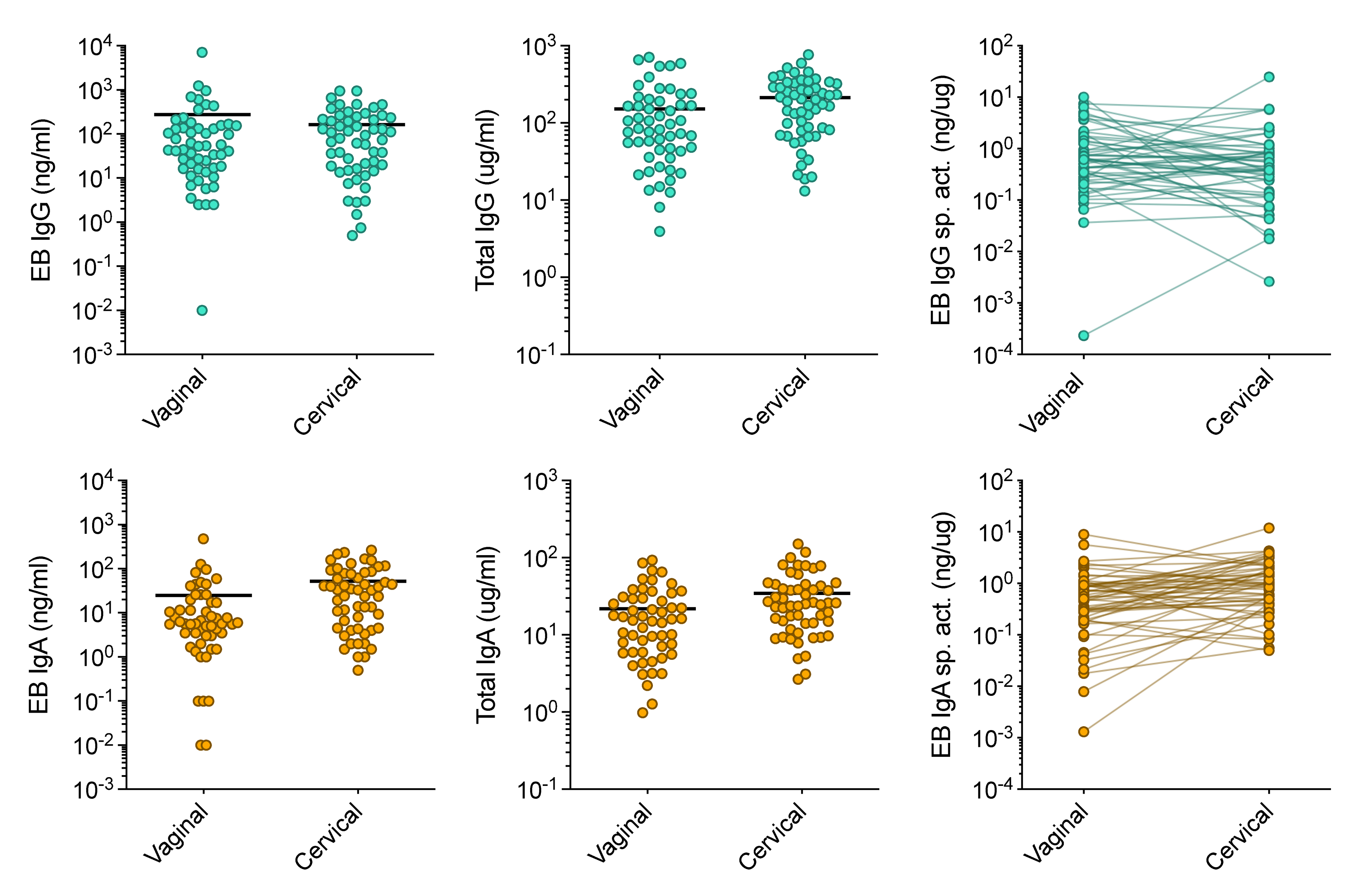

Supplement: S1 Fig — EB specific activity was calculated by dividing Ct-specific antibody by total immunoglobulin for each isotype and sampling site (ng anti-EB IgG or IgA antibody per μg total IgG or IgA, respectively). (TIF) [file pone.0258759.s002.tif]
